# Plasma metabolomic profiles differ at the time of artificial insemination based on pregnancy outcome, in *Bos taurus* beef heifers

**DOI:** 10.1038/s41598-018-31605-0

**Published:** 2018-09-04

**Authors:** Kaitlyn M. Phillips, Casey C. Read, Lisa A. Kriese-Anderson, Soren P. Rodning, Terry D. Brandebourg, Fernando H. Biase, M. Landon Marks, Joshua B. Elmore, M. Kent Stanford, Paul W. Dyce

**Affiliations:** 1 Department of Animal Sciences, College of Agriculture, Auburn University, Auburn, AL 36849 USA; 2 Alabama Cooperative Extension System, Auburn, AL USA

## Abstract

Infertility remains the most prevalent reason for cattle being removed from production environments. We utilized metabolomic profiling to identify metabolites in the blood plasma that may be useful in identifying infertile heifers at the time of artificial insemination (AI). Prior to AI, phenotypic parameters including body condition, weight, and reproductive organ measurements were collected. These were determined not effective at differentiating between fertile and infertile heifers. Analysis of the resulting metabolomic profiles revealed 15 metabolites at significantly different levels (T-test P ≤ 0.05), with seven metabolites having a greater than 2-fold difference (T-test P ≤ 0.05, fold change ≥2, ROC-AUC ≥ 0.80) between infertile and fertile heifers. We further characterized the utility of using the levels of these metabolites in the blood plasma to discriminate between fertile and infertile heifers. Finally, we investigated the potential role inflammation may play by comparing the expression of inflammatory cytokines in the white blood cells of infertile heifers to that of fertile heifers. We found significantly higher expression in infertile heifers of the proinflammatory markers tumor necrosis factor alpha (*TNFα)*, interleukin 6 *(IL6)*, and the C-X-C motif chemokine 5 (*CXCL5*). Our work offers potentially valuable information regarding the diagnosis of fertility problems in heifers undergoing AI.

## Introduction

Unexplained infertility remains a significant source of inefficiency within the cow calf production sector. The ability to identify heifers with high reproductive potential for recruitment into the breeding stock is one of the keys to efficient cattle production. Currently, due to a lack of accurate biomarkers, replacement heifers are selected based on phenotypic and genetic background information^1,2^. Analysis of breeding data involving 3,144 records found pregnancy rates varied between locations from 75–95% in Angus heifers^3^. Poor fertility accounts for the majority of cows culled and remains largely unmanageable due to a lack of informative biomarkers^4^.

The underlying causes leading to this persistent population of infertile heifers remain largely unknown. Recently the role of the immune system has been implicated in playing important roles in fertility. Altered inflammatory cytokine expression in the endometrium has been seen in heifers with failing pregnancies including the higher expression of interleukin 6 (*IL-6*), interleukin 10 (*IL-10*), and interferon α (*INFA*)^5^. Furthermore, in cattle, inflammation has been shown to perturb the growth and steroidogenesis of the preovulatory follicle and negatively impact conception rates^6–8^. In fact, bovine granulosa cells have been shown to express functional Toll-like receptors (TLRs) able to respond to lipopolysaccharide (LPS) and Pam3CSK4 (PAM) exposure leading to the upregulation of proinflammatory cytokines^6^. Currently, the specific mechanism responsible for inflammatory responses effecting reproductive performance in cattle remains to be well defined.

Metabolomics, or metabolomic profiling, involves the quantitative measurement of the global set of low molecular weight metabolites in a biological fluid^9^. Metabolite levels can then be compared between different phenotypic states and potentially be used as health indicators. Mass spectrometry-based metabolite analysis has been applied in the development of many informative biomarkers to help identify hard to diagnose disorders^10–13^. Recent studies have utilized metabolomic analysis in assessing embryo and oocyte quality^14–16^. Moreover, within cattle, metabolomic analysis of follicular fluid has been used to potentially explain differences in fertility between heifers and lactating cows^17^. Authors in that study found metabolites at significantly different levels, when comparing heifers to cows, in the blood serum and follicular fluid^17^. While the differences in metabolite levels between heifers and cows could be related to fertility differences, it is also possible that they are due to age. Plasma metabolomic profiling has also proven effective at identifying potential biomarkers for metabolic disorders such as ketosis, in dairy cattle^18–20^. Furthermore, metabolomic approaches have been extensively used within the biomedical field to develop biomarkers aimed at diagnosing difficult to diagnose pathologies. However, to our knowledge, there is a lack of studies looking into the relationship between metabolomic profiles and reproductive outcomes in cattle.

As such, in the present study, we conducted comprehensive metabolomic profiling of the blood plasma of heifers at the time of artificial insemination (AI). Samples were analyzed via untargeted profiling of primary metabolism by automatic linear exchange/cold injection gas chromatography time-of-flight mass spectrometry (GC-TOF-MS). Heifers were AIed and then 14 days later placed with a fertile bull for three consecutive estrous cycles. Metabolomic profiles were compared between heifers that became pregnant from the AI and those that remained open following the breeding season. In addition, to better characterize the potential role of inflammation, we analyzed the expression levels of proinflammatory cytokines in the white blood cells of heifers with differing pregnancy outcomes following AI and natural breeding.

## Results

### Phenotypic heifer assessment

**Figure 1:**
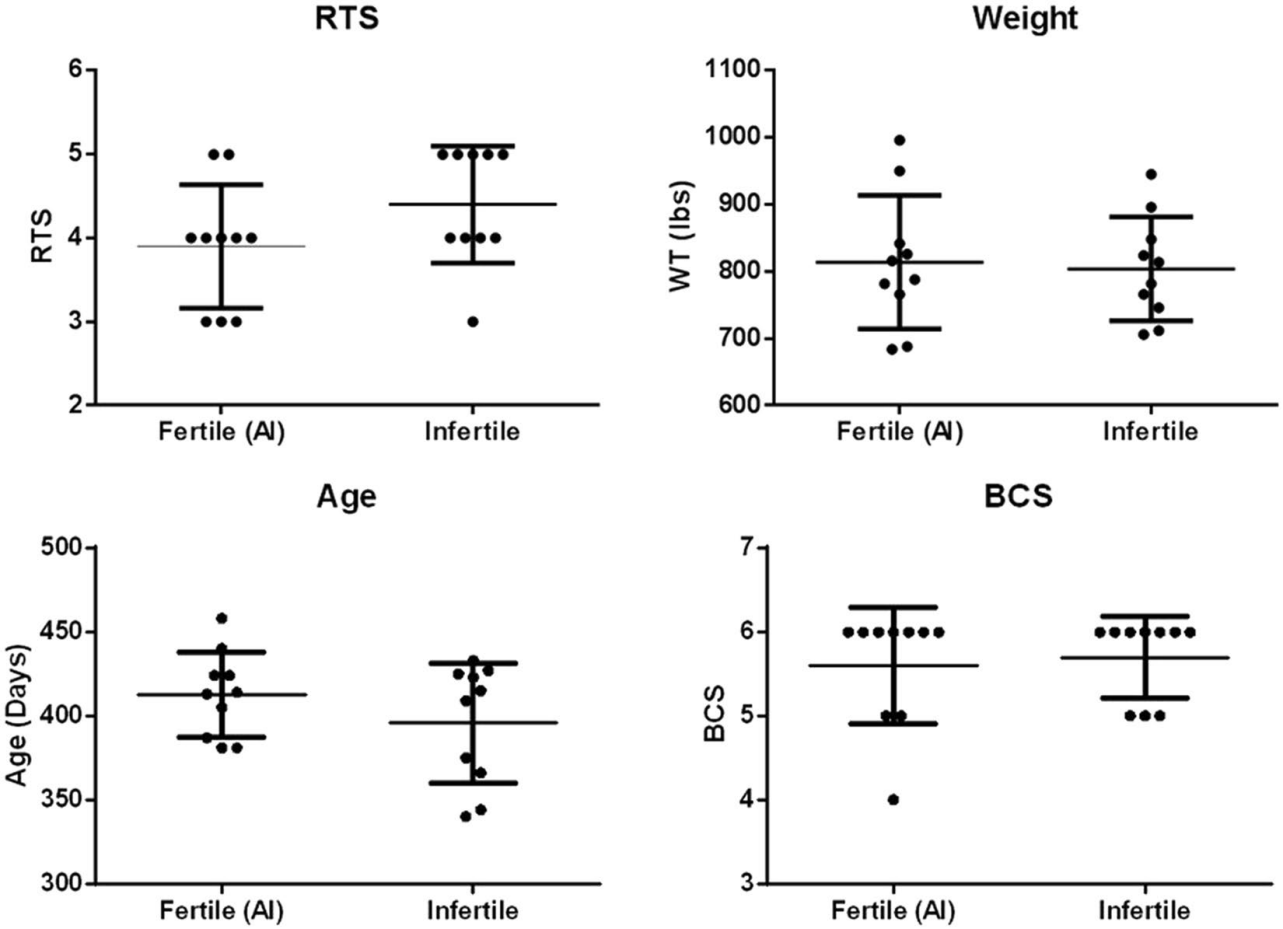
Phenotypic comparisons between fertile (N = 10) and infertile (N = 10) heifers. No significant difference was seen in Reproductive tract scores (RTSs), weight at AI (WT), age at AI (Age), or body condition scores (BCS) between heifers becoming pregnant by artificial insemination and those remaining open (T-test, P > 0.05).

In order to determine if accessible phenotypic differences could differentiate between fertile and infertile heifers, we collected reproductive tract score (RTS), weight at AI, age at AI, and body condition score (BCS). No significant difference was seen in RTSs between heifers becoming pregnant by artificial insemination (AI; 3.9 ± 0.74) or those remaining open (4.4 ± 0.70)(P > 0.05, Fig. 1). Weight (WT) at AI was also found to be not significantly different between heifers becoming pregnant by AI (813.8 ± 99.6 lbs.) or those remaining open (803.9 ± 77.56 lbs.)(P > 0.05, Fig. 1). Heifer age at AI was not significantly different between heifers pregnant by AI (412.7 ± 25.4 days) or those remaining open (395.7 ± 35.9 days)(P > 0.05, Fig. 1). No significant differences were seen in BCSs between AI pregnant heifers and those remaining open, 5.6 ± 0.7 and 5.7 ± 0.5 respectively (P > 0.05, Fig. 1).

### Metabolites identified as differentially expressed

**Figure 2:**
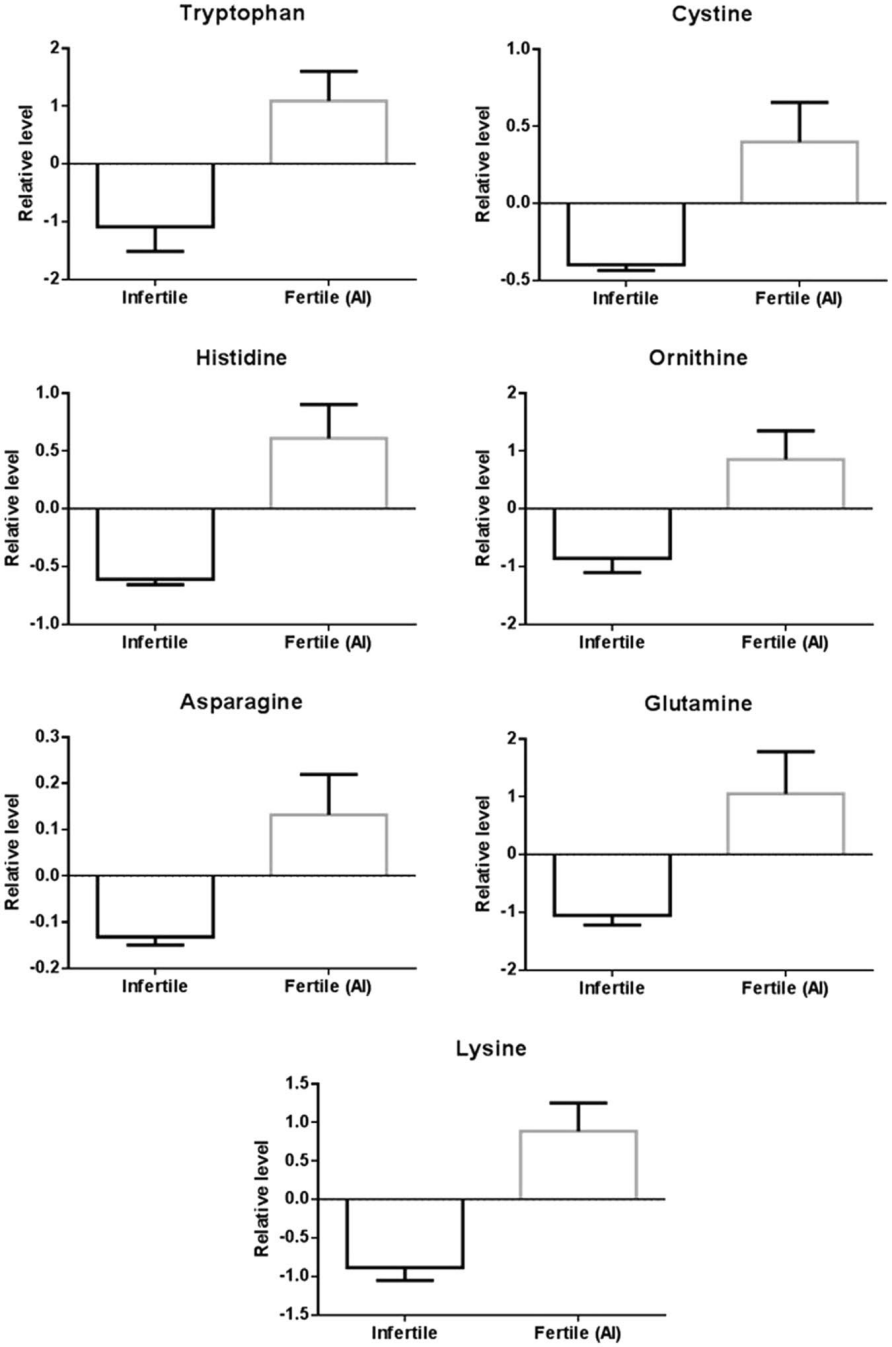
Relative levels of marker metabolites identified at significantly different levels (>2-fold, P < 0.05, FDR < 0.05) in infertile heifers (N = 10) when compared to fertile heifers (N = 10).

**Figure 3:**
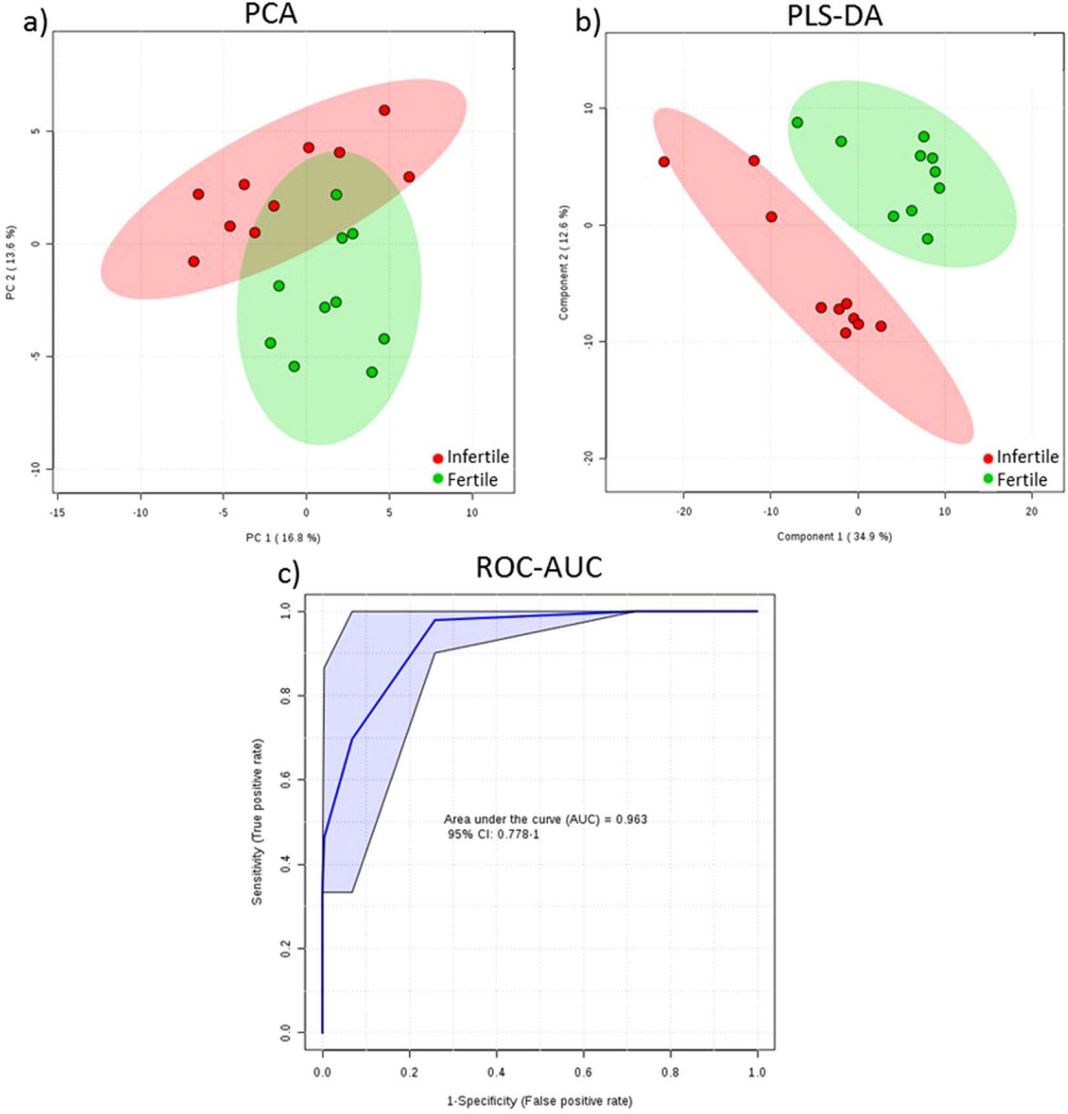
PCA, PLS-DA, and AUC-ROC analysis score plots. (**a**) PCA of the infertile (N = 10, red) and fertile (N = 10, green) samples depicting separation. (**b**) PLS-DA analysis scores plots showing significant separation (P = 0.05 by permutation test) between infertile (N = 10, red) and fertile (N = 10, green) heifers. (**c**) ROC curve of fertile and infertile heifers at the time of AI.

**Figure 4:**
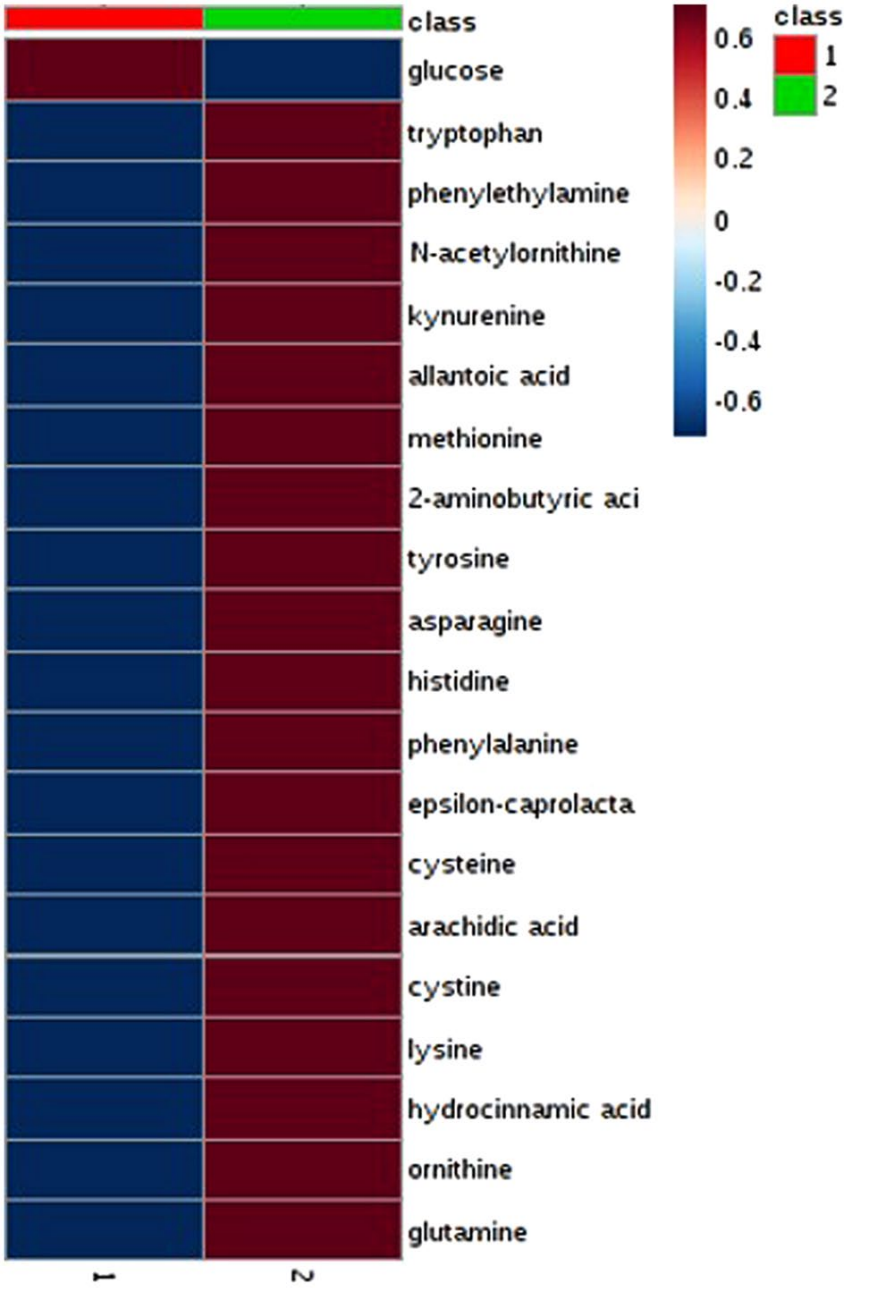
Heat map depicting top 20 metabolites at different levels based on T-test results. Samples were grouped as infertile red (N = 10, 1) and fertile green (N = 10, 2) heifers.

Univariate T-test analysis found 15 significantly different metabolite levels between the fertile and infertile plasma samples (Table 1). Following filtering for metabolites with at least a 2-fold change between samples, tryptophan, cystine, histidine, ornithine, asparagine, glutamine, and lysine were identified as significantly different (P < 0.05, FDR 0.05) between the fertile and infertile groups (Table 1, Fig. 2). PCA and PLS-DA analysis displayed group separation between fertile and infertile samples (Fig. 3). The top twenty metabolites at differential levels, as identified via T-test, displayed a trend of being down regulated in the infertile heifers (Fig. 4).

**Table 1:** Metabolites found at significantly different levels in the infertile heifers when compared to the fertile heifers.

Metabolite	P-value	Fold Change	log2 (FC)	ROC-AUC
Asparagine	0.009	0.484	−1.047	0.89
Lysine	0.001	0.445	−1.167	0.89
Ornithine	0.006	0.428	−1.225	0.85
Glutamine	0.010	0.358	−1.483	0.94
Histidine	0.001	0.189	−2.403	0.91
Cystine	0.007	0.145	−2.787	0.80
Tryptophan	0.004	0.512	−0.965	0.86
Hydrocinnamic acid	0.004	0.526	−0.926	0.86
2-aminobutyric acid	0.013	0.550	−0.863	0.87
Cysteine	0.047	0.596	−0.747	0.74
Phenylethylamine	0.020	0.602	−0.732	0.83
Methionine	0.014	0.612	−0.709	0.87
Kynurenine	0.018	0.631	−0.665	0.80
N-acetylornithine	0.023	0.644	−0.634	0.79
Allantoic acid	0.037	0.665	−0.589	0.75

### Predictive ability of metabolites identified

In order to classify the predictive ability of the identified metabolites (P < 0.05, >2 fold change), we calculated the Receiver Operating Characteristic (ROC) area under the curve (AUC) value. The ROC AUC values were 0.86 for tryptophan, 0.80 for cystine, 0.91 for histidine, 0.85 for ornithine, 0.89 for asparagine, 0.93 for glutamine, and 0.87 for lysine (Table 1). Twenty new samples were randomly selected and used to produce metabolimic profiles. Metabolmic profiles generated from the new samples, including 13 fertile and 7 infertile heifers, were interrogated with the previously identified metabolites individually, and in combination, to validate their potential use as biomarkers for fertility. Metabolites with the highest ROC AUC value in the logistical regression model were glutamine (0.91), asparagine (0.89), and histidine (0.88). We tested the models on the blood plasma metabolomes of the 20 heifers to validate their potential use as biomarkers for fertility. Glutamine and histidine alone, and in combination, predicted the correct pregnancy outcome in 90% of the animals and did not incorrectly categorize a fertile animal as infertile (Table 2).

**Table 2:** ROC-AUC analysis of metabolites found to be at different levels in infertile heifers compared to fertile heifers (AUC (95% CI)).

Metabolite Tested	AUC	Sensitivity	Specificity	% correctly categorized	% fertile categorized as infertile
Tryptophan	0.820 (0.625–1.000)	0.800 (0.800–1.000)	0.700 (0.416–0.984)	80	10
Cystine	0.720 (0.456–0.984)	0.700 (0.700–0.984)	0.800 (0.552–1.000)	70	15
Histidine	0.880 (0.727–1.000)	0.700 (0.700–0.984)	1.000 (1.000–1.000)	90	0
Ornithine	0.840 (0.664–1.000)	0.800 (0.800–1.000)	0.700 (0.416–0.984)	70	0
Asparagine	0.890 (0.694–1.000)	0.900 (0.900–1.000)	0.900 (0.714–1.000)	70	0
Glutamine	0.910 (0.773–1.000)	0.800 (0.800–1.000)	1.000 (1.000–1.000)	90	0
Lysine	0.860 (0.659–1.000)	0.800 (0.800–1.000)	0.900 (0.714–1.000)	75	0
His, Glut, Asp	0.735 (0.465–1.000)	0.800 (0.800–1.000)	0.900 (0.714–1.000)	85	0
His, Glut	0.860 (0.681–1.000)	0.900 (0.900–1.000)	0.800 (0.552–1.000)	90	0

### Pathway analysis

Following the adjustment of p values (FDR < 0.05) the aminoacyl-tRNA biosynthesis pathway was found to be significantly affected (Table 3).

**Table 3:** Pathway Analysis.

	Total	Expected	Hits	Raw p	−log(p)	Holm adjusted p value
Aminoacyl-tRNA biosynthesis	64	0.690	7	1.51E-6	13.401	0.0001
Cysteine and methionine metabolism	28	0.301	3	0.003	5.868	0.2264
Nitrogen metabolism	9	0.097	2	0.004	5.588	0.2957
Arginine and proline metabolism	44	0.474	3	0.010	4.575	0.8037
Alanine, aspartate and glutamate metabolism	23	0.248	2	0.024	3.725	1
Glutathione metabolism	26	0.280	2	0.030	3.494	1
D-Glutamine and D-glutamate metabolism	5	0.05	1	0.053	2.940	1
Biotin metabolism	5	0.054	1	0.053	2.940	1
Tryptophan metabolism	41	0.442	2	0.070	2.661	1
Taurine and hypotaurine metabolism	7	0.075	1	0.073	2.614	1
Thiamine metabolism	7	0.075	1	0.073	2.614	1
Phenylalanine metabolism	9	0.097	1	0.093	2.373	1
Histidine metabolism	14	0.150	1	0.141	1.956	1
Pantothenate and CoA biosynthesis	15	0.162	1	0.150	1.892	1
Purine metabolism	68	0.733	2	0.164	1.806	1
Glycine, serine and threonine metabolism	32	0.345	1	0.296	1.218	1

### Inflammation

**Figure 5:**
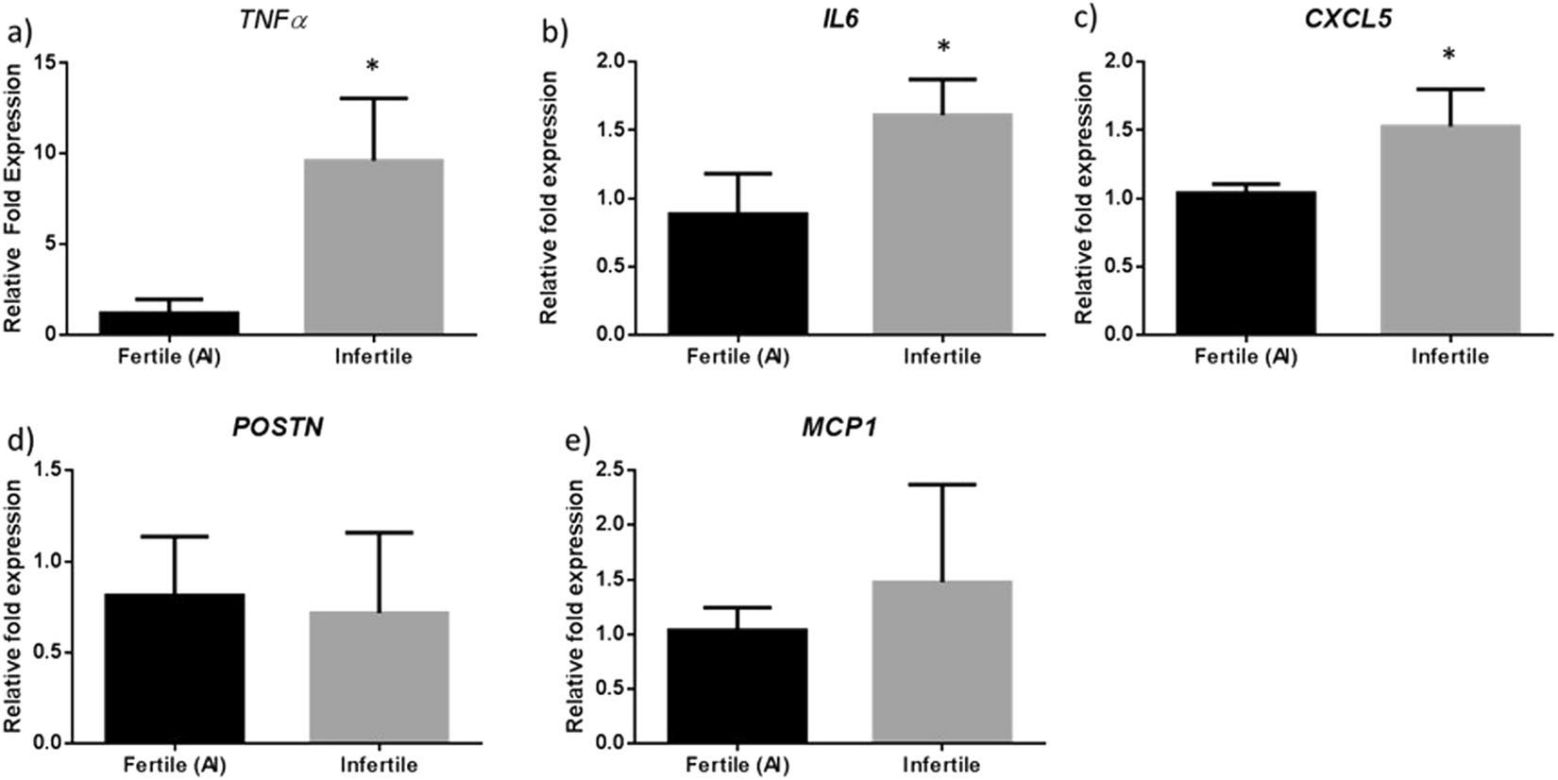
Comparison of transcript levels of inflammatory cytokines in white blood cells from fertile (N = 3) and infertile heifers (N = 3). *TNFα*, IL6, and CXCL5 were expressed at a significantly lower level in the fertile heifers when compared to the infertile heifers. POSTN and MCP1 were found to be expressed at similar levels in the fertile and infertile heifers (P = 0.77 and P = 0.37, respectively). *Denoted significant results (T-test, P < 0.05).

In order to investigate the possibility that infertile heifers have asymptomatic inflammation, we isolated the mRNA from white blood cells and compared the transcript level of inflammatory cytokines to fertile heifers. The transcript level of the inflammatory response regulator tumor necrosis factor alpha (*TNFα*) was found to be significantly higher in infertile heifers (P = 0.003, 9.62 ± 3.43 fold), when compared to fertile heifers (1.22 ± 0.76 fold) (Fig. 5a). Similarly, transcripts for the pro-inflammatory cytokine interleukin 6 (*IL-6*) were found to be significantly higher in the infertile heifers (P = 0.01, 1.61 ± 0.26 fold), when compared to fertile heifers (0.89 ± 0.30 fold) (Fig. 5b). We also found the neutrophil activating peptide C-X-C motif chemokine 5 (*CXCL5*) to be significantly higher in the infertile heifers (P = 0.04, 1.53 ± 0.27 fold), when compared to the fertile heifers (1.04 ± 0.07 fold) (Fig. 5c). Conversely, we did not see a significant difference in the expression of the interleukin induced Periostin (*POSTN*) gene when we compared infertile (P = 0.77, 0.72 ± 0.44 fold) to fertile heifers (0.81 ± 0.32 fold) (Fig. 5d). Moreover, we did not see a difference in the expression of the pro-inflammatory chemokine monocyte chemoattractant protein 1 (*MCP1*) when we compared the infertile (P = 0.37, 1.48 ± 0.45 fold) to the fertile heifers (1.04 ± 0.21 fold) (Fig. 5e).

## Discussion

The main goal of our study was to generate comparative metabolomic profiles at the time of AI between fertile and infertile heifers. We identified heifers that became pregnant from AI and those that failed to become pregnant following the breeding season. Blood was sampled from heifers housed at three locations over the course of two breeding seasons. Heifers did not differ with respect to age at AI, RTS, BCS, or weight. It is important to note that all of the heifers included in the study had a RTS of ≥3, supporting them being pubescent during the breeding season^21^. Furthermore, heifers included in the study did not differ with respect to age or BCS further supporting the inability to differentiate these heifers based on current phenotypic traits. Using this system, we were able to categorize heifers with differing fertility potentials, which is consistent with previous studies^22–25^. A previous study compared Holstein heifers with a BCS below the median to those above the median with regards to their rate of conception at first service^26^. They did not see a significant correlation between BCS and ability to become pregnant at first service^26^. In our study, we only included heifers with a BCS deemed acceptable. While there is no question both RTS and BCS are useful metrics for evaluating replacement heifers, they remain somewhat subjective and are unable to detect a portion of problematic breeders.

Utilizing metabolomic analysis of the blood plasma of heifers becoming pregnant at first service through AI and those failing to become pregnant following AI, and three natural breeding cycles, we identified 15 metabolites present at significantly different levels. Furthermore, we identified seven metabolites that had a greater than 2-fold difference between the two groups. The seven identified metabolites had ROC AUC values ranging from 0.80–0.94, suggesting that they can predict fertility problems at the time of AI better than chance. We then used each of the identified metabolites individually, and in combination, to develop a logistic regression model to predict pregnancy outcomes of new samples. We found the percentage of animals correctly categorized ranged from 70% using asparagine, ornithine, or cysteine to 90% using glutamine or histidine. Interestingly, utilizing histidine in combination with glutamine did not improve the detection of infertile heifers with the accuracy remaining at 90%. All other combinations or individual metabolites did not perform as well. Particularly problematic is the labelling of fertile heifers as infertile. This would run the risk of removing potentially valuable animals as a result of a false diagnosis. Using histidine or glutamine individually or in combination did not result in any fertile heifers being identified as infertile. In all cases of false identification, we observed infertile heifers being categorized as fertile.

Pathway analysis revealed a significant effect on the aminoacyl-tRNA biosynthesis pathway. This is not surprising as 7 out of the 15 metabolites shown to be at different levels in the infertile heifer’s blood plasma, were amino acids. The 7 amino acids were all present at a lower level in the infertile heifers when compared to the fertile group. In our study, we found lower levels of asparagine, lysine, glutamine, histidine, tryptophan, cysteine, and ornithine. Previously, the differential levels of amino acids in the blood of dairy cattle have been shown to correlate with ketosis^18,19,27^. One study looking at dairy cows with clinical ketosis found differential levels of 13 metabolites^19^. Among the metabolites, 7 were amino acids. Similar to our study, they found decreased lysine and tryptophan in cattle showing clinical signs of ketosis^19^. Not surprisingly, they found a relationship between glucogenic amino acids such as arginine, valine, and glycine which are processed into phosphoenolpyruvate to synthesize glucose, and clinical ketosis^28^. They also found a correlation between clinical ketosis and the levels of ketogenic amino acids such as leucine and lysine, which can be converted to ketones in the liver^29^, and amino acids that are both glucogenic and ketogenic such as tryptophan and isoleucine^19,30^. These studies support amino acid levels in the blood plasma being dynamic and reflecting the metabolic status of the animal.

The majority of studies in cattle looking at infection and reproductive performance have utilized dairy cattle and focused on the common postpartum complication mastitis^31^. A potential mechanism and link between inflammation and infertility in cattle has been proposed by Hansen *et al*.^32^ involving inflammation caused by bacterial infection leading to increased proinflammatory cytokine production effecting levels of reproductive hormones, increasing body temperature, and destabilizing the corpus luteum^32^. In fact, mastitis has been shown to decrease reproductive parameters including conception rate^33^. Using metabolomics, a recent study compared the plasma metabolomic profiles between dairy cows with subclinical mastitis and healthy control cows^34^. They identified 17 metabolites, with good predictive abilities, altered in the subclinical population during the week of diagnosis including valine, serine, tyrosine, and phenylalanine^34^. In a companion study they found that cattle with subclinical mastitis had greater serum concentrations of the proinflammatory cytokine TNFα^35^, suggesting the initiation of systemic infection preceding clinical mastitis. In both studies, the effect of inflammation and subsequent mastitis on reproductive performance was not investigated. Metabolomics has been used to investigate the relationship between inflammation and the amino acid profiles in the blood plasma in different species. One study where we did see similar metabolomic profiles looked at the levels of amino acids in the blood plasma of kidney disease patients with and without inflammation^25^. They found those with inflammation had significantly lower levels of asparagine, serine, glutamine, glycine, arginine, alanine, histidine, and threonine^36^. Similarly, in another study investigating the plasma amino acid levels of cats with chronic gastrointestinal (GI) disease and associated inflammation, it was shown that arginine, histidine, lysine, methionine, phenylalanine, taurine, and tryptophan (along with several non-essential amino acids) were lower^37^. Furthermore, they found that histidine could suppress inflammatory cytokine release by their macrophages^37^. Therefore, the decreased level of histidine found in the infertile heifer’s blood plasma was of particular interest due to its well described antioxidant and anti-inflammatory functions^37,38^. Studies have shown the presence of low plasma histidine is associated with inflammation^39^. Furthermore, studies in humans have shown supplementation with histidine suppressed inflammatory cytokines such as TNFα and IL6^40^. Interestingly, it has been shown that histidine supplementation can affect the levels of other metabolites in the blood, leading to increased glutamine, aspartate, glycine, choline, and trimethylamine-*N*-oxide in humans^41^. In our study, we also observed decreased glutamine, along with histidine, in the infertile heifers. We therefore investigated the expression of various inflammatory cytokines in the white blood cells from the fertile and infertile heifers. We observed a significant upregulation in the transcripts for the inflammatory cytokines *TNFα*, *IL6*, and *CXCL5*, suggesting a potential relationship between fertility and the inflammatory status in heifers. The specifics of this relationship remains to be studied.

In conclusion, we utilized metabolomics to investigate the differential levels of metabolites in the blood plasma of fertile and infertile heifers. We identified 15 metabolites with differential levels between the two groups. The predictive ability of the metabolites was assessed and used to predict pregnancy outcomes at the time of AI. Furthermore, infertile heifers had significantly higher expression of *TNFα*, *IL-6*, and *CXCL5* in their white blood cells. Whether the levels of these metabolites are present at different levels, at a younger age, is a current focus of ongoing research. It is acknowledged that the number of animals utilized in this study were limited, and that validation in a larger cohort of animals in the future is warranted.

## Materials and Methods

### Animal use

All procedures involving animals were approved by the Auburn University Institutional Animal Care and Use Committee (IACUC) and conform with Planning Research and Experimental Procedures on Animals: Recommendations for Excellence (PREPARE) and Animal Research: Reporting of *In Vivo* Experiments (ARRIVE) guidelines. Heifers utilized for this study (N = 167) originated from and were housed at the Black Belt, Wiregrass, Gulf Coast, and Sand Mountain Research and Extension Centers located throughout Alabama, U.S.A. as part of the Alabama Agricultural Experiment Station. Selected heifers at all locations were placed on pasture (fescue/bermuda grass) from weaning until calving with free-choice ryegrass hay available. All heifers received soyhull/corn-gluten mixture supplementation and trace minerals ad libitum. Angus and Angus-cross heifers underwent an estrus synchronization and fixed-time artificial insemination program (7-day CO-Synch + CIDR^42^), spanning two breeding seasons 2015/16 and 2016/17. Briefly, at the initiation of the estrus synchronization protocol, all heifers received 100 µg GnRH via intramuscular injection (CYSTORELIN^®^, Merial Animal Health, Duluth, GA, USA), and a controlled internal drug release (CIDR) device containing 1.38 g of progesterone was placed intravaginally (EAZI-BREED CIDR Cattle Insert, Zoetis, Kalamazoo, MI, USA). Each CIDR was removed following 7 days, and an intramuscular injection of 25 mg of dinoprost tromethamine (LUTALYSE, Zoetis, Kalamazoo, MI, USA) was administered at the same time. Heifers were then artificially inseminated with a single straw of semen originating from selected Angus sires 54 ± 2 h following CIDR removal. A second intramuscular injection of 100 µg GnRH was administered at the time of artificial insemination (AI). Fourteen days following AI, heifers were exposed to an intact sire for three consecutive estrous cycles. Bulls at each research facility were all proven breeders. All bulls passed a standard BSE (Breeding Soundness Exam) with semen quality having <10% abnormality, and all were cleared for any reproductive discrepancies for each breeding season. Bulls were placed at an average density of 1 bull per 33 heifers for 60 days following artificial insemination.

### Phenotypic observations

A total of 56 (n = 56) heifers were used, split between two breeding seasons (2 years), and were analyzed for phenotypic conditions including body condition score (BCS), reproductive tract score (RTS), and weight at AI (WT). BCS was determined as previously described^43^. The BCS scale ranged from 1–9, with 1 being emaciated and 9 being obese. Only heifers with an ideal body condition score of 4–6 were included in this study. Reproductive tract score (RTS) evaluation was performed by veterinarians by transrectal palpation. Heifers were assigned a RTS ranging from 1–5 based on uterine size, uterine tone, ovarian size, and ovarian structure, as previously described^21^. Briefly, heifers with immature uterine horns (<20 mm in diameter) and lacking muscle tone, with no palpable ovarian follicles were scored as a 1. Heifers with 20–25 mm in diameter uterine horns with no tone, and ≤8 mm ovarian follicles were scored as a 2. Heifers with similar sized uterine horns but with some tonality and follicles measuring 8–10 mm were scored a 3. Heifers with uterine horns between 25–30 mm in diameter showing good tone and >10 mm ovarian follicles or corpus luteum were scored as a 4. Finally, heifers with >30 mm in diameter uterine horns with good tone and clear corpus luteum present were given a score of 5. The BCS and RTS were collected one month prior to AI.

### Heifer selection for metabolomic analysis

Twenty heifers (10 AI-pregnant and 10 non-pregnant) were selected taking representatives from each research station for metabolomic analysis. Heifers were selected based on their similarities in age and phenotypic characteristics. All heifers included had reached puberty and were similar in age and weight.

### Blood collection and processing

At the time of artificial insemination, 10 ml of blood was collected via jugular vein of each heifer using an 18G needle into an EDTA blood collection tube (BD Vacutainer). The sample was immediately inverted 10 times, placed on ice, and transported to the lab. Once in the lab, samples were centrifuged at 2,000 × *g* for 15 min at 4 °C. Two 500 µl samples of blood plasma were removed and stored at −80 °C for metabolomic analysis. Samples were stored at −80 °C until further processing.

### Pregnancy determination

Pregnancy was determined at 45 and 65 days post AI via transrectal palpation by a trained veterinarian. Heifers were identified as pregnant (AI), pregnant (Bull) or non-pregnant based on the size of the conceptus. In this study only samples from heifers remaining open, following the AI and natural breeding exposure (infertile), and those impregnated through AI (fertile) were analyzed for metabolite levels.

### Metabolomic data collection

Blood plasma samples collected at the time of AI (N = 10 pregnant by AI and N = 10 non-pregnant) were used to identify metabolites at different levels. A further 20 blood plasma samples collected at the time of AI (N = 13 pregnant by AI and N = 7 non-pregnant) were used to determine the predictive potential of the metabolites found at different levels in the first sample set. Samples (N = 40) had metabolomic profiles generated via untargeted profiling of primary metabolism by automatic linear exchange/cold injection GC-TOF-MS on a Agilent 6890 GC equipped with a Gerstel automatic liner exchange system (ALEX) and a cold injection system (Gerstel, Muehlheim, Germany). Injection volume was 0.5 μl with 10 μl/s injection speed. Datum was acquired with the following chromatographic parameters: column used Rtx-5Sil MS (30 m X 0.25 mm diameter Restek corp.) with a 0.25 µm 95% dimethyl/5% diphenylpolysiloxane film; mobile phase Helium with a 1 ml/min flow rate; injection volume 0.5 µl^44^. The oven temperature is held constant at 50 °C for 1 min and then ramped at 20 °C/min to 330 °C at which it is held constant for 5 min. Electron impact ionization was at 70 V and employed with an ion source temperature of 250 °C. Raw data files were preprocessed directly using ChromaTOF 2.32. Absolute spectra intensities were further processed by a filtering algorithm implemented in the metabolomics BinBase database. Quantification is reported as peak height using the unique ion as default. Binned data was normalized and scaled to remove potential bias arising due to sample handling and variability. Normalization by sum was performed followed by scaling (mean-centering and division by the square root of standard deviation of each variable), to give all variables equal weight regardless of their absolute value.

### Univariate statistical analysis

Univariate analysis was applied to a total of 122 identified metabolites consistently present in all plasma samples from 10 fertile (pregnant by AI) and 10 infertile (open). Data was normalized by sum in order to minimize variations introduced by sample extraction. Following normalization, scaling (mean-centering and division by the square root of standard deviation of each variable) was performed to equally weigh each variable regardless of absolute value. T tests were performed with an FDR cutoff of 0.05. Metabolites were considered at significantly different levels when P < 0.05.

### Multivariate statistical analysis

Multivariate analysis was applied to a total of 122 metabolites from 10 fertile and 10 infertile plasma samples. Data was normalized by sum in order to minimize concentration differences. Following normalization, scaling (mean-centering and division by the square root of standard deviation of each variable) was performed to equally weigh each variable regardless of absolute value. Partial Least Squares Discriminant Analysis (PLS-DA) was then performed using MetaboAnalyst (accessible at http://metaboanalyst.ca)^45^ using functions from the R and Bioconductor packages^46^ in order to maximize class discrimination. Model robustness was assessed using Receiver operating characteristic – Area Under Curve (ROC-AUC) analysis using MetaboAnalyst software. Classification models were built based on metabolites showing significant differential levels (T-test, FDR < 0.05) with at least a 2-fold difference. Twenty blinded samples were used to test the robustness of the models used to characterize heifers as fertile and infertile. Further validation was performed with MetaboAnalyst using permutation tests.

### Metabolic pathway analysis

Metabolic Pathway Analysis was performed using MetaboAnalyst 3.0. Pathway Analysis combined results from Pathway Enrichment Analyses’ and Pathway Topology Analyses’ to correctly identify relative pathways involved in both fertile and infertile samples. Parameters for Metabolic Pathway Analysis included normalization by sum and Pareto data scaling (mean-centered and divided by the square root of the standard deviation) of each variable presented. KEGG-metabolic pathways were utilized to determine the course of each individual metabolite.

To analyze the effect of 15 differential metabolites identified on biological pathways, a Fisher’s Exact Test was performed.

### Buffy coat isolation

Samples were centrifuged in an EDTA blood collection tube at 2,000 × *g* for 15 min at 4 °C to separate plasma and buffy coat layers. Following centrifugation, a 500 µl band of buffy coat was aseptically pipetted and re-suspended into a sterile 15 ml centrifuge tube containing 12 ml ice cold lysis solution (0.15 mM ammonium chloride, 10 µM sodium bicarbonate, and 1.3 µM EDTA). Tubes were inverted every two minutes for a total of ten minutes. Tubes were then centrifuged at 250 × *g* for 10 min at 4 °C in order to form a visible pellet. The supernatant was discarded, and pellets were gently re-suspended in a 1.5 ml micro-centrifuge tube containing 1 ml wash buffer (PBS with 2% fetal bovine serum). Samples were centrifuged in a tabletop centrifuge at 250 × *g* for 10 minutes at 4 °C. The supernatant was discarded, and pellets were stored at −80 °C until further processing.

### RNA isolation and cDNA synthesis

Total buffy coat RNA was isolated from the pelleted sample using the illustra^TM^ RNAspin Mini RNA Isolation Kit (GE Healthcare, Buckinghamshire, UK) following the manufacturer’s instructions. Samples were subjected to DNase treatment for 15 min at room temperature. The RNA was then analyzed using a Qubit Fluorometer (Thermo Fisher Scientific). One µg of isolated RNA was then reverse transcribed (RT) into cDNA using qScript cDNA Supermix (Quanta BioSciences Inc., Beverly, MA).

### Real-time PCR

To account for the variations in RNA concentrations, C_q_ values of the samples were normalized to the C_q_s of the reference gene GAPDH. GAPDH presented similar C_q_ values across all samples (P = 0.59, T-test). For fertile and infertile samples, four total isolations were used. The isolated RNA was then reverse transcribed (RT) to cDNA using qScript cDNA supermix (Quanta BioSciences Inc., Beverly, MA) according to the manufacturer’s recommended protocol. Primers for *GAPDH*, *TNFα*, *IL6*, *CXCL5, POSTN*, and *MCP1* were validated for product specificity and efficiency tested prior to use (Supplementary Table S1). A Roche LightCycler 480 Real-time qPCR machine was utilized to compare the expression levels of the target transcripts using the delta-delta C_q_ method and *GAPDH* was used as an internal loading control^47^. The averages of gene expression levels were considered statistically different when P ≤ 0.05. The qPCR reactions were run using PerfeCTa SYBR Green Supermix (Quanta Biosciences Inc., Beverly, MA) according to the manufacturer’s protocol.

## Electronic supplementary material

Supplementary Table S1

## Data Availability

The datasets generated and analyzed during the current study are available from the corresponding author on reasonable request.
