# Supplementary material for: Plasma metabolomic profiles differ at the time of artificial insemination based on pregnancy outcome, in Bos taurus beef heifers
Source: Sci Rep. 2018 Sep 4;8:13196. doi: 10.1038/s41598-018-31605-0 (PMC6123494; doi:10.1038/s41598-018-31605-0)
Supplement: Supplementary file 1 — Supplementary Table S1 [file 41598_2018_31605_MOESM1_ESM.docx]

Supplementary Table S1

Title: Plasma metabolomic profiles differ at the time of artificial insemination based on pregnancy outcome, in *Bos taurus* beef heifers.

Kaitlyn M Phillips^1^, Casey C Read^1^, Lisa A Kriese-Anderson^1^, Soren P Rodning^1^, Terry D Brandebourg^1^, Fernando H Biase^1^, Landon Marks^2^, Joshua B. Elmore^2^, Kent Stanford^2^, and Paul W Dyce^1*^

^1^Department of Animal Sciences, College of Agriculture, Auburn University, Auburn, AL 36849, USA

^2^Alabama Cooperative Extension System, Auburn, AL, USA

Supplementary Table S1: Primer Design

| **Primer** | **Sequence**  **(5🡪 3’)** | **NCBI Accession Number** | **Efficiency (%)** | **Product Length (bp)** |
| --- | --- | --- | --- | --- |
| **TNFα** | F: TCAAGCCTCAAGTAACAAGCC  R: GTTGTCTTCCAGCTTCACACC | NM_173966.3 | 93 | 123 |
| **IL6** | F: TGAGTGTGAAAGCAGCAAGGA  R: TCGCCTGATTGAACCCAGAT | NM_173923.2 | 100 | 100 |
| **CXCL5** | F: AAAGTTGCCCAGTTCTTCAG  R: CAAGCATAGATTCCCTCTTCC | BC142108.1 | 95 | 146 |
| **POSTN** | F: TGTGTTATATGAATGCTGCCCT  R: ATCCCTTTCCTTCAATCTCCTC | AY445072.2 | 91 | 169 |
| **MCP1** | F: CTCAGCCAGATGCAATTAACTC  R: AAATCACAGCCTCTTTAGGAC | NM_174006.2 | 91 | 128 |
| **GAPDH** | F: CGTAACTTCTGTGCTGTGCC  R:ATTGATGGCGACGATGTCCA | NM_001034034.2 | 107 | 136 |
